# Correlates of cannabis use in a sample of mental health treatment-seeking Canadian armed forces members and veterans

**DOI:** 10.1186/s12888-023-05237-2

**Published:** 2023-11-14

**Authors:** Kate St. Cyr, Anthony Nazarov, Tri Le, Maede Nouri, Priyonto Saha, Callista A Forchuk, Vanessa Soares, Sonya G. Wanklyn, Brian M. Bird, Brent D. Davis, Lisa King, Felicia Ketcheson, J. Don Richardson

**Affiliations:** 1 MacDonald Franklin OSI Research Centre, Lawson Health Research Institute, London, ON Canada; 2 Dalla Lana School of Public Health, University of Toronto, Toronto, ON Canada; 3 Dept. of Psychiatry, Western University, London, ON Canada; 4 Dept. of Psychiatry & Behavioural Neuroscience, McMaster University, Hamilton, ON Canada; 5 St. Joseph’s OSI Clinic, St. Joseph’s Health Care London, London, ON Canada; 6 Peter Boris Centre for Addictions Research, McMaster University, Hamilton, ON Canada

**Keywords:** Cannabis, Mental health, Military personnel, Veterans

## Abstract

**Objective:**

Canadian Armed Forces (CAF) members and Veterans are more likely to experience mental health (MH) conditions, such as posttraumatic stress disorder (PTSD), than the general Canadian population. Previous research suggests that an increasing number of individuals are employing cannabis for MH symptom relief, despite a lack of robust evidence for its effectiveness in treating PTSD. This research aimed to: (1) describe the prevalence of current cannabis use among MH treatment-seeking CAF members and Veterans; and (2) estimate the association between current cannabis use and a number of sociodemographic, military, and MH-related characteristics.

**Method:**

Using cross-sectional intake data from 415 CAF members and Veterans attending a specialized outpatient MH clinic in Ontario, Canada, between January 2018 and December 2020, we estimated the proportion of CAF members and Veterans who reported current cannabis use for either medical or recreational purposes. We used multivariable logistic regression to estimate adjusted odds ratios for a number of sociodemographic, military, and MH-related variables and current cannabis use.

**Results:**

Almost half of the study participants (n = 187; 45.1%) reported current cannabis use. Respondents who reported current cannabis use for medical purposes had a higher median daily dose than those who reported current cannabis use for recreational purposes. The multivariable logistic regression identified younger age, lower income, potentially hazardous alcohol use, and increased bodily pain as statistically significant correlates of current cannabis use among our MH treatment-seeking sample. PTSD severity, depressive severity, sleep quality, and suicide ideation were not statistically associated with current cannabis use.

**Conclusions:**

Almost half of our treatment-seeking sample reported current cannabis use for medical or recreational purposes, emphasizing the importance of screening MH treatment-seeking military members and Veterans for cannabis use prior to commencing treatment. Future research building upon this study could explore the potential impact of cannabis use on MH outcomes.

## Introduction

Previous research indicates that Canadian Armed Forces (CAF) members and Veterans are more likely to experience mental health (MH) conditions than members of the general Canadian population [1, 2]. Of the CAF Regular Force Veterans who completed the 2013 Life After Service Survey, 17.1% (95% CI = 15.4–19.0) self-reported a mood disorder diagnosis, 13.1% (95% CI = 11.6–14.8) self-reported a PTSD diagnosis, and 11.1% (95% CI = 9.7–12.7) self-reported an anxiety disorder diagnosis [3]; and many Veterans will experience two or more MH conditions concurrently. Indeed, mental health and well-being surveys commissioned by the CAF and Veterans Affairs Canada (VAC) for active-duty military personnel and Veterans have shown that the presence of comorbid mental disorders has increased between 2002 and 2018 [4]. The presence of complex comorbidities, such as substance use and depressive disorders, creates unique challenges in the treatment of military-related PTSD.

While a number of pharmacologic and psychotherapeutic guidelines for PTSD exist [5], previous research suggests that Veterans may continue to experience MH symptoms even after treatment with evidence-based trauma-focused therapies [6, 7]. Treatment efficacy may be further reduced among Veterans with PTSD and one or more psychiatric comorbidities [8, 9]. As such, it is possible that a proportion of Veterans do not experience sufficient abatement of their symptoms through first-line psychotherapeutic or pharmacologic interventions and instead turn to cannabis for symptom relief, with or without a prescription [10], while others may potentially employ cannabis before or instead of engaging in first-line treatment as a means of coping with their MH symptoms. Indeed, the number of Canadian Veterans using cannabis for medical purposes has increased dramatically within the past decade, with VAC providing reimbursements for medical cannabis amounting to over $150,000,000 in the 2021–2022 fiscal year [11]. This may be partially attributable to legislative changes made in 2014 [12] which increased access to cannabis for medical purposes, and 2018 [13], which increased access to cannabis for all Canadian adults, regardless of the purpose for use (i.e., recreational or medical). With this increase in the availability of and access to cannabis, Veterans experiencing complex MH symptoms may be particularly likely to engage in cannabis use for symptom management.

However, despite the growing use of cannabis for MH symptom management, there is a paucity of high-quality empirical evidence supporting the notion that cannabis imparts beneficial effects on PTSD symptoms. Within Veteran populations, existing evidence is mixed. In a study of Veterans with PTSD who remained symptomatic following a course of conventional psycho- or pharmacotherapy, cannabis use was associated with improvements across a range of common PTSD symptoms, such as anger and irritability, anxiety, avoidance, and depersonalization [13]. Conversely, other research suggests that heavy cannabis use is associated with an increased risk of depressive disorders and other adverse MH effects, ranging from mild (e.g., lightheadedness) to severe (e.g., paranoia and suicide behaviours) [14–16]. Further, a recent systematic review by O’Neil and colleagues concluded that there is insufficient evidence to determine whether cannabis is effective in alleviating symptoms associated with common MH disorders, across a range of populations, including Veterans [17]. The lack of robust evidence for the use of cannabis in the management of military-related PTSD symptoms, combined with increasing rates of utilization among Canadian Veterans, warrants further investigation into the characteristics of MH treatment-seeking CAF members and Veterans, in order to identify individuals who may have an increased likelihood of using cannabis alongside other conventional MH therapies. This descriptive study aims to: (1) estimate the prevalence of cannabis use within a consecutive MH treatment-seeking sample of CAF members and Veterans; and (2) evaluate the association between select sociodemographic, military-, and health-related characteristics and current cannabis use among a sample of MH treatment-seeking CAF members and Veterans residing in Ontario, Canada.

## Methods

### Study design, participants, and setting

The current study utilized cross-sectional, self-reported data collected at intake from English-speaking CAF members and Veterans referred to the St. Joseph’s Operational Stress Injury (OSI) Clinic in London, Ontario, an outpatient MH clinic that specializes in assessing and treating PTSD and other service-related MH conditions. Participants completed all study measures between January 2018 and December 2020.

All measures were administered as part of a standardized intake protocol, and data were de-identified and stored in an electronic database. At intake, all participants provided written informed consent for their information to be used for research purposes. Institutional and ethical approval was received from the Lawson Health Research Institute and Western University’s Health Sciences Research Ethics Board (approval # 113374), respectively.

### Measures

#### Outcome variable

Cannabis use was measured using two self-report items: (1) “Do you use medical cannabis (If yes, how many grams per day)?”; and (2) “Do you use recreational cannabis (If yes, how many grams per day)?”. Because of concerns around the number of events-per-variable included in the regression models, participants were categorized as either using cannabis currently for any reason or not currently using cannabis. There was a notable amount of missing data for the question pertaining to daily dosage; as such, these data were not included in regression analyses; however, descriptive data related to dosage are presented.

#### Covariates

Demographic variables included sex (dichotomous; male or female), age at intake (dichotomous; <40 years and ≥ 40 years), military status (dichotomous; Veteran or still-serving member), highest level of education achieved (categorical; completed high school or less vs. some college or university vs. completed college or university), household income (dichotomous; <$60 000 to ≥$60 000), and marital status (dichotomous; married/common-law vs. separated/divorced/widowed/single [never married]).

PTSD symptoms were assessed using the PTSD Checklist-5 (PCL-5) [18], a 20-item, self-report measure assessing symptoms of PTSD using DMS-5 criteria [19]. Respondents were asked to indicate how bothered they had been by each item over the past month using a scale where 0 = “Not at all,” and 4 = “Extremely.” Responses were summed to provide an overall score ranging from 0 to 80, where higher scores indicated greater symptom severity. A score of 33 or higher is considered indicative of probable PTSD. In a validation study of Veterans, the PCL-5 demonstrated good internal consistency (α = 0.96) and test-retest reliability (r = 0.84) [20]. The internal consistency of the PCL-5 in the current study was also high (α = 0.90).

Suicide ideation was measured using a single item from the Patient Health Questionnaire-9 (PHQ-9) [21] that read, “Over the past two weeks, how often have you been bothered by thoughts that you would be better off dead, or hurting yourself in some way?”. This item has been used in previous research [22–24] as an indicator of suicide ideation, and was associated with suicide risk among patients in a Veterans Health Administration (VHA) setting [25]. For this study, we dichotomized responses to this item such that participants who did not report any past two-week suicide ideation were coded as 0 (“No”) while those who reported any suicide ideation in the past two weeks, regardless of frequency, were coded as 1 (“Yes”).

The remaining eight items of the PHQ-9 were used to assess depressive symptom severity. Respondents indicated how frequently they had experienced each depressive symptom over the past two weeks on a scale where 0 = “Not at all” and 3 = “Nearly every day”. To account for the removal of the suicide ideation item, responses were summed to provide a total score ranging from 0 to 24; higher scores indicate higher levels of depressive symptom severity. Used as an eight-item measure, the PHQ has slightly reduced sensitivity compared to the PHQ-9 but similar specificity [26]. Internal consistency in the current study was excellent (α = 0.94).

Alcohol use disorder (AUD) was measured using the Alcohol Use Disorder Identification Test (AUDIT) [27], a ten-item self-report measure assessing current alcohol use. Responses ranged from 0 (“Never”) to 4 (“4 or more times a week”) for drinking frequency; 0 (“None”) to 4 (“10 or more”) for drinking quantity; 0 (“Never”) to 4 (“Daily or almost daily”) for drinking consequences; and 0 (“No”), 2 (:Yes, but not in the past year”), or 4 (“Yes, during the past year”) for concern expressed by others and alcohol-related injury. Potential scores ranged from 0 to 40 with scores of eight or more indicating probable alcohol misuse. The AUDIT has good sensitivity and specificity, and has been used across a variety of populations, including military populations [28, 29]. In the current study, the internal consistency of the AUDIT was acceptable (α = 0.66).

The two-item “Bodily Pain” subscale of the Medical Outcomes Questionnaire – 36-Item Short Form Survey (SF-36) [30] was used to evaluate current bodily pain severity and pain interference. Possible scores range from 0 to 100, with lower scores representing increased pain disturbances.

Sleep quality was measured using the Pittsburgh Sleep Quality Index (PSQI) [31]. The PSQI consists of seven components, including duration of sleep, sleep disturbance, sleep latency, daytime dysfunction due to sleepiness, sleep efficiency, overall sleep quality, and use of medication to sleep, each of which are scored from 0 to 3. A total score ranging from 0 to 21 is generated, with higher scores indicating poorer sleep quality. The PSQI demonstrated acceptable internal consistency in a sample of Veterans with severe PTSD (α = 0.78) [32], and good sensitivity (89.6%) and specificity (86.5%) in initial validation studies [31]. The internal consistency of the PSQI in the current study was high (α = 0.88).

### Data Analysis

The proportion of study participants reporting any current cannabis use was calculated. Sociodemographic and clinical characteristics of the study sample were described by current cannabis use status using means and standard deviations for continuous variables, and frequencies and percentages for categorical variables.

Data preprocessing was done to remove individuals who did not respond to the questions related to cannabis use or who had missing values for more than 50% of the intake questionnaire items (n = 8; 1.9% of initial sample). The open-ended item 5j on the PSQI (“How often have you had trouble sleeping because of other reasons?”) was missing significant amounts of data. As recommended by the scale’s authors, we imputed zeroes for any missing comments or values on this question, while missing data for any other MH scale item were handled using the multivariate imputation by chained equations (MICE) procedure in R. For the MICE procedure, eight imputed datasets with five iterations were used. Fully conditional specification was used for MH item imputation, with scale totals computed in each imputed dataset.

PTSD, depression, suicide ideation, alcohol use, bodily pain, and sleep quality scores were plotted to visually assess for deviations from normality using histograms. Bivariate associations between cannabis use status and MH symptom severity scores were assessed using unadjusted binary logistic regression models.

Finally, multivariable logistic regression models were used to estimate the adjusted odds ratios (AORs) of sociodemographic, military-, and health-related variables associated with current cannabis use. The first model included only sociodemographic and military-related variables. The second model additionally included MH-related variables (e.g., symptom severity scores). Likelihood ratio tests were used to evaluate model fit, with the second model outperforming the first. As such, only output from the second model is presented here.

We estimated 97.5% confidence intervals (CIs) for the regression models, and assumed a statistically significant association for *p*-values of less than 0.05. All analyses were conducted using R version 4.1.3.

## Results

### Sample characteristics

Data was collected from a total of 415 CAF members and Veterans (see Table 1). The average age of the sample was 45.6 (SD = 12.7) years. Most of the participants were male (80.2%, n = 333) and were either married or in a common-law relationship (56.6%, n = 235). In addition, most of the participants were Veterans of the CAF (92.3%, n = 383).

**Table 1:** Sociodemographic and clinical characteristics of the study sample prior to imputation, overall and by current cannabis use status

Variable	Overall (n = 415)	Cannabis use (n = 187)	No cannabis use (n = 228)
	*Mean (SD)*		
Age	45.6 (12.7)	43.0 (11.6)	47.8 (13.2)
PCL-5 score	44.6 (18.5)	46.4 (18.7)	43.0 (18.2)
PHQ-8 score	15.4 (6.1)	16.2 (5.8)	14.8 (6.3)
AUDIT score	6.4 (7.6)	7.8 (8.7)	5.3 (6.5)
PSQI score	12.6 (4.1)	13.0 (3.8)	12.4 (4.2)
SF-36 bodily pain	39.6 (23.8)	34.9 (23.1)	43.4 (23.7)
	*n (%)*		
Sex			
Male Female Missing	333 (80.2) 73 (17.6) 9 (2.2)	154 (82.4) 29 (15.5) 4 (2.1)	179 (78.5) 44 (19.3) 5 (2.2)
Marital status			
Married/common law Single/divorced/widowed Missing	235 (56.6) 173 (41.7) 7 (1.7)	105 (56.2) 79 (42.2) 3 (1.6)	130 (57.0) 94 (41.2) 4 (1.8)
Education			
Completed college Some college Completed high school or less Missing	160 (38.6) 129 (31.1) 121 (29.2) 5 (1.2)	72 (38.5) 67 (35.8) 47 (25.1) 1 (0.5)	88 (38.6) 62 (27.2) 74 (32.5) 4 (1.8)
Military status			
CAF Veteran Still serving CAF member	383 (92.3) 32 (7.7)	174 (93.0) 13 (7.0)	209 (91.7) 19 (8.3)
Income			
<$60,000 ≥$60,000 Don’t know Missing	149 (35.9) 232 (55.9) 23 (5.5) 11 (2.7)	82 (43.9) 93 (49.7) 10 (5.4) 2 (1.1)	67 (29.4) 139 (61.0) 13 (5.7) 9 (3.9)
Any past 2-week suicide ideation			
Yes No Missing	182 (43.9) 229 (55.2) 4 (1.0)	87 (46.5) 99 (52.9) 1 (0.5)	95 (41.7) 130 (57.0) 3 (1.3)

### Cannabis use

A total of 187 (45.1%) participants reported current cannabis use. A larger proportion of individuals who reported current cannabis use were male compared to those who did not report current cannabis use (82.4% vs. 78.5%), and fell into the <$60 000/year income category compared to individuals who did not report current cannabis use (43.9% vs. 29.4%). A slightly higher proportion of individuals who reported current cannabis use were Veterans compared to those who did not report current use (93.0% vs. 91.7%). A similar proportion in each cannabis use status group reported having completed post-secondary school (38.5% and 38.6%), but a higher proportion of individuals in the no current use group had completed high school or less compared to those in the current cannabis use group (32.5% vs. 25.1%).

**Table 2:** Self-reported daily cannabis dosage, in grams, by current cannabis use type

	Mean (SD)	Median (IQR)	Range
Cannabis for medical purposes (n = 105)	2.8 (1.6)	3.0 (1)	< 0.1 to 10.0
Cannabis for recreational purposes (n = 94)	2.2 (3.6)	1.0 (1.6)	< 0.1 to 20.0
Cannabis for both medical and recreational purposes (n = 12)^a,b^	4.3 (2.9)	3.5 (4.4)	1.0 to 10.0

^a^Values for individuals who reported current medical and recreational use were derived by summing the reported daily doses for medical and recreational purposes; ^b^medical and recreational doses from individuals who reported both medical and recreational use are also contained within the “cannabis for medical purposes” and “cannabis for recreational purposes” rows, respectively

SD = standard deviation; IRQ = interquartile range

Twelve respondents reported using cannabis for both medical and recreational purposes; their reported average daily dosages were higher than individuals who reported current use for either medical or recreational purposes alone (i.e., 4.3 g vs. 2.8 g and 2.2 g, respectively; see Table 2). The median daily dose reported by individuals who reported current cannabis use for medical or combined medical/recreational purposes was higher than the median dose reported by individuals who endorsed current cannabis use for recreational purposes (i.e., 3.0 g vs. 1.0 g).

### Factors associated with current cannabis use

**Table 3:** Adjusted odds ratios of sociodemographic, military, and health-related variables associated with current cannabis use

Variable	AOR (97.5% CI)	*p*-value
Age (ref = 40 + years)		
** <40 years**	**2.00 (1.28–3.12)**	< 0.01*
Sex (ref = female)		
Male	1.07 (0.61–1.88)	0.81
Marital status (ref = separated/divorced/ single)		
Married/common-law	1.42 (0.88–2.28)	0.15
Education (ref = completed college/ university)		
High school or less Some college/university	0.78 (0.46–1.32) 1.25 (0.75–2.09)	0.35 0.39
Annual household income (ref = ≥ $60 000)		
** <$60 000**	**1.85 (1.13–3.03)**	0.02*
Veteran status (ref = Veteran)		
Still serving CAF member	1.28 (0.56–2.89)	0.56
PCL-5 total score	1.00 (0.98–1.01)	0.72
PHQ-8 total score	1.02 (0.97–1.07)	0.48
**AUDIT total score**	1.06 (1.02–1.09)	< 0.01*
PSQI total score	0.99 (0.92–1.06)	0.78
Suicide ideation (ref = no)		
Yes	0.92 (0.57–1.29)	0.74
**SF-36 bodily pain subscale**	**0.98 (0.97–0.99)**	< 0.01*

*AOR = adjusted odds ratio; PCL-5 = PTSD Checklist; PHQ-8 = Patient Health Questionnaire (excluding suicide ideation item); AUDIT = Alcohol Use Disorder Identification Test; PSQI = Pittsburgh Sleep Quality Inventory; ref = reference group; SF-36 = MOS Short Form Health Survey-36*

In the multivariable model (Table 3**)**, the odds of current cannabis use were twice as high for individuals under the age of 40 than individuals who were 40 years of age or older (AOR = 2.00; 97.5% CI = 1.28–3.12), while having an annual household income below $60 000 was associated with an 85% increase in the odds of current cannabis use (AOR = 1.85; 97.5% CI = 1.13–3.03). Current cannabis use was also statistically associated with higher AUDIT scores (AOR = 1.06; 97.5% CI = 1.02–1.09), and pain, such that a one-point increase in the SF-36 bodily pain subscale (indicating less severe pain) was associated with a 2% decrease in the odds of current cannabis use (AOR = 0.98; 97.5% CI = 0.97–0.99).

## Discussion

In our sample of MH treatment-seeking CAF members and Veterans, almost half (45.1%) reported current cannabis use. Respondents who reported current cannabis use for medical purposes reported a higher median daily dose than those who reported current cannabis use for recreational purposes (i.e., 3 g vs. 1 g), which may be related to VAC reimbursement amount policies (up to 3 g of cannabis daily for Veterans with a medical authorization) [33]. Individuals reporting current cannabis use were more likely to be younger, have a lower income (an annual household income of <$60,000), endorse more problematic alcohol use behaviours, and report higher levels of bodily pain than individuals who reported no current cannabis use. These findings are consistent with existing research related to cannabis use within Veteran populations. Previous research has reliably demonstrated a relationship between younger age and increased cannabis use/cannabis use disorders within Veteran populations [34–36]. Further, a recent study exploring correlates of medical and recreational cannabis use among US Veterans receiving primary care at a VHA clinic found that being younger and lower income were both associated with increased likelihood of past-year cannabis use [37], while a wide body of literature has documented increased likelihood of cannabis use among Veterans with chronic pain [37–39]. The observed association between cannabis use status and increased alcohol use echoes findings from numerous research reports [37, 40–44], and adds to the mounting evidence related to the co-occurrence of cannabis and alcohol use. Further research aimed at disentangling potential differences in alcohol misuse by type of cannabis use (i.e., recreational, medical, or both) among MH treatment-seeking Veterans would provide important information that could be used to guide treatment-making decisions.

Interestingly, in the multivariable model, none of the other MH symptom severity measures investigated in our study, including PTSD symptoms, depressive symptoms, or sleep quality, were statistically associated with cannabis use status. These findings echo some of the findings of an earlier study of 120 CAF Veterans with PTSD, which found that approximately 50% of participants reported cannabis use, and that no association between cannabis use status and PTSD symptom severity was observed [45]. Other studies have similarly reported non-significant findings in terms of the association between PTSD symptom severity and cannabis use status [40, 42]. However, the non-significant associations between PTSD and depressive symptom severity and cannabis use observed in this study contrast other existing research [37, 46], which found statistically significant associations between meeting diagnostic criteria for PTSD and cannabis use. The mixed findings pertaining to the association between psychiatric disorders and cannabis use may be due to differences in study samples; indeed, the null findings observed in the current study may be at least partially attributable to the MH treatment-seeking nature of our study sample (i.e., all participants were initiating care related to an operational stress injury and were equally apt to be highly symptomatic at the time of data collection), and the accessibility of cannabis to Canadians during the study period. It is notable that participants responses to the PCL-5 in this study were not necessarily anchored to a Criterion A event but rather a “stressful experience”, which may partially explain inconsistent findings.

This study has several strengths. First, it builds upon existing research by surveying MH treatment-seeking Canadian military members and Veterans about their current cannabis use, and provides an estimate of the proportion of MH treatment-seeking CAF members and Veterans who report current cannabis use for either medical or recreational purposes. This information helps quantify the proportion of treatment-seeking Canadian Veterans who use cannabis, as data from VAC records may not include Veterans who receive their cannabis for medical purposes elsewhere, and does not include Veterans who use cannabis recreationally. This research may also help military and Veteran health researchers to identify further opportunities to study cannabis use among Canadian Veterans longitudinally, and in a non-observational manner (i.e., randomized trials to better elucidate the effectiveness of cannabis for military-related PTSD).

The findings of the current study are limited by their cross-sectional nature, in that we cannot tell whether higher symptom severity drives cannabis use, or whether cannabis use exacerbates existing MH symptoms. However, while the association between PTSD and cannabis use was not statistically significant in this study, the associations between cannabis use and (a) substance use and (b) bodily pain were, meaning it is possible that a subset of Veterans use cannabis for symptom abatement in the presence of complex MH comorbidities. The item used to ascertain current cannabis use status did not include a specified time (i.e., “within the past thirty days”), potentially reducing the precision of the prevalence estimate reported in this study. Additionally, it is possible that the prevalence of cannabis use described in our study is specific to MH treatment-seeking samples, and should not be generalized to other non-treatment-seeking samples. Finally, this study provides only a preliminary glance into cannabis use among outpatient MH treatment-seeking CAF members and Veterans. Future research in this area should consider the influence of additional variables, such as length and frequency of cannabis use, modes of cannabis administration, cannabinoid concentrations, motivation for use, and historical and concurrent conventional and alternative treatment, which may influence the role of cannabis use on MH outcomes.

These findings have important clinical and health policy implications. First, the relatively high proportion of participants who reported cannabis use for medical or recreational purposes highlights the importance of screening treatment-seeking military and Veteran populations for cannabis use prior to commencing treatment. It is possible that MH professionals working with military populations are in a unique position to offer psychoeducation about the potential risks and benefits of cannabis use; and clinicians should carefully consider the potential implications of cannabis use on treatment planning, delivery, and outcomes. These findings may also help form an evidence base for Veteran cannabis-related health policies related to treatment practice guidelines and VAC-funded cannabis.

## Data Availability

The data used in this study are not publicly available for access, as they are part of patient health records, but requests for de-identified data may be made to Dr. J. Don Richardson at don.richardson@sjhc.london.on.ca.

## Abbreviations

AOR: adjusted odds ratio
AUD: alcohol use disorder
AUDIT: Alcohol Use Disorder Identification Test
CAF: Canadian Armed Forces
CI: confidence interval
MH: mental health
MICE: multivariable imputation by chained equations
OSI: operational stress injury
PCL5: PTSD Checklist–5
PHQ-9: Patient Health Questionnaire–9
PTSD: posttraumatic stress disorder
PSQI: Pittsburgh Sleep Quality Inventory
SF-36: Medical Outcomes Questionnaire–36–Item Short Form Survey
VAC: Veterans Affairs Canada
VHA: Veteran Health Administration
